# Performance Evaluation of Warm-Mix Asphalt Binders with an Emphasis on Rutting and Intermediate-Temperature Cracking Resistance

**DOI:** 10.3390/ma18071571

**Published:** 2025-03-30

**Authors:** Jiangbo Pang, Yu Chen, Longfei Jing, Haoran Song, Ziyang Liu

**Affiliations:** 1 Baiquan Branch of Shaanxi Transportation Holding Group Co., Ltd., Ankang 725000, China; pang_198010@163.com (J.P.); j_19880713@163.com (L.J.); 2 School of Highway, Chang’an University, Middle Section of NanErhuan Road, Xi’an 710064, China; g12315@126.com (H.S.); 2024021076@chd.edu.cn (Z.L.)

**Keywords:** warm-mix asphalt, additives, rotational viscosity, rutting resistance, cracking resistance

## Abstract

Warm-mix asphalt (WMA) technology is gaining popularity worldwide due to its benefits of considerable emissions reduction and energy savings when compared with hot-mix asphalt (HMA). Currently, there is a wide range of WMA products with considerable variability in the corresponding pavement performances. It has also been difficult to reach a unified conclusion regarding the effects of various WMA additives on asphalt binder properties. In this study, two categories of warm-mix additives, including six organic additives and three chemical additives, were evaluated in terms of their effects on asphalt binder properties, with a focus on rutting and intermediate-temperature cracking. It was found that the viscosity-reducing effect of organic additives was more significant in comparison to chemical additives. In addition, the binders modified with the organic additives obtained enhanced rutting resistance at high temperatures but compromised cracking resistance at intermediate temperatures, as shown by the increasing complex modulus (G*) and non-recoverable creep compliance (*J_nr_*) and decreasing binder fracture energy (BFE). Meanwhile, the very limited effect of chemical additives on rutting resistance was observed while the cracking resistance was slightly improved. The findings will assist in the selection and application of WMA additives for the production of asphalt mixture.

## 1. Introduction

Warm-mix asphalt (WMA) technology has been increasingly utilized in asphalt pavement construction due to its distinct benefits, including reduced mixing temperature, decreased energy consumption, attenuation of the high-temperature aging of asphalt during construction, effective prolongation of the service life of the asphalt pavement, and protection of the health of construction workers. Currently, there are three main WMA technologies, including organic additives, chemical additives, and water-foaming technology [1,2,3]. There is quite a variety of WMA products available on the market, and there is considerable variability in their corresponding pavement performances, as the latter depends largely on the type of warm-mix technology used, as well as the type and dosage of additives [2].

Due to the melting of wax at temperatures above melting point, organic additives can have a very good viscosity-reducing effect during the production process [4,5,6,7]. A lattice structure [8] was also reported to be created below the melting point of Sasobit (an organic additive); this increased its resistance to deformation and thus improved its rutting resistance [6,7,8,9]. In addition, Deurex (an organic additive) has been reported to have better rutting resistance in terms of softening point, failure temperature, stiffness, and storage modulus than Sasobit [9]. However, the stiffness and physical hardening of asphalt binders are increased due to the crystallization and precipitation of waxes [10], which significantly compromises their low-temperature cracking resistances. As stated earlier, the type of wax, its chemical structure, and its melting point play a significant role in asphalt binder properties. For instance, Fischer–Tropsch and fatty acid amide waxes have been reported to be effective in improving rutting and fatigue resistance, whereas paraffin wax has an overall negative effect [11]. Asphalt mixtures prepared with Sasobit exhibited higher stiffness and fatigue cracking resistance than foaming technology [12]. Also, the Fischer–Tropsch paraffin exhibited better rutting resistance than polyethylene wax-modified asphalt mixtures based on a French rutting test [13].

Chemical additives (surfactant-based warm-mix additives) act at the microscopic interface between the aggregates and asphalt binders, reducing internal friction forces and thus increasing the workability and compactability for asphalt mixtures at lower temperatures [14]. Evotherm M1 (a chemical additive) can improve aggregate-binder adhesion and thus reduce binder viscosity, which leads to lower mixing and compaction temperatures of 30–50 °C [15]. Evotherm M1 was reported to have a better viscosity-reduction effect than Retherm (a chemical additive), and both were able to effectively enhance the lifespan of high-viscosity asphalt [16]. Meanwhile, the deformation resistance of high-viscosity binders was increased in both Evotherm M1 and Retherm, but the improvement effect of Evotherm M1 was higher than that of Retherm [16]. On the other hand, Rediset (a chemical additive) was found to reduce the rutting potentials of asphalt binder in terms of non-recoverable creep compliance (*J_nr_*), while Sasobit was reported to increase the rutting resistance of asphalt binders [17,18]. Rediset was also revealed to enhance the fatigue resistance as compared with pure and Sasobit-modified asphalt binder [18]. Wu et al. [19] reported that after field aging, asphalt binders with Evotherm are still comparable to conventional HMA binders based on binders extracted from field samples.

It can be concluded that WMA additives from different categories and different additives from the same category have different effects on both asphalt binders and asphalt mixture performances. Thus, it is worth further investigating the effects of different WMA additives on asphalt binder performances. In this study, the effects of two categories of warm-mix additives, comprising six organic additives and three chemical additives, on the performance of asphalt binders at both high and intermediate temperatures are thoroughly examined. The results reveal the action mechanisms of the two categories of additives on asphalt and will assist in the selection and application of WMA additives for the production of asphalt mixtures in the future.

## 2. Materials and Methods

### 2.1. Materials

**Figure 1:**
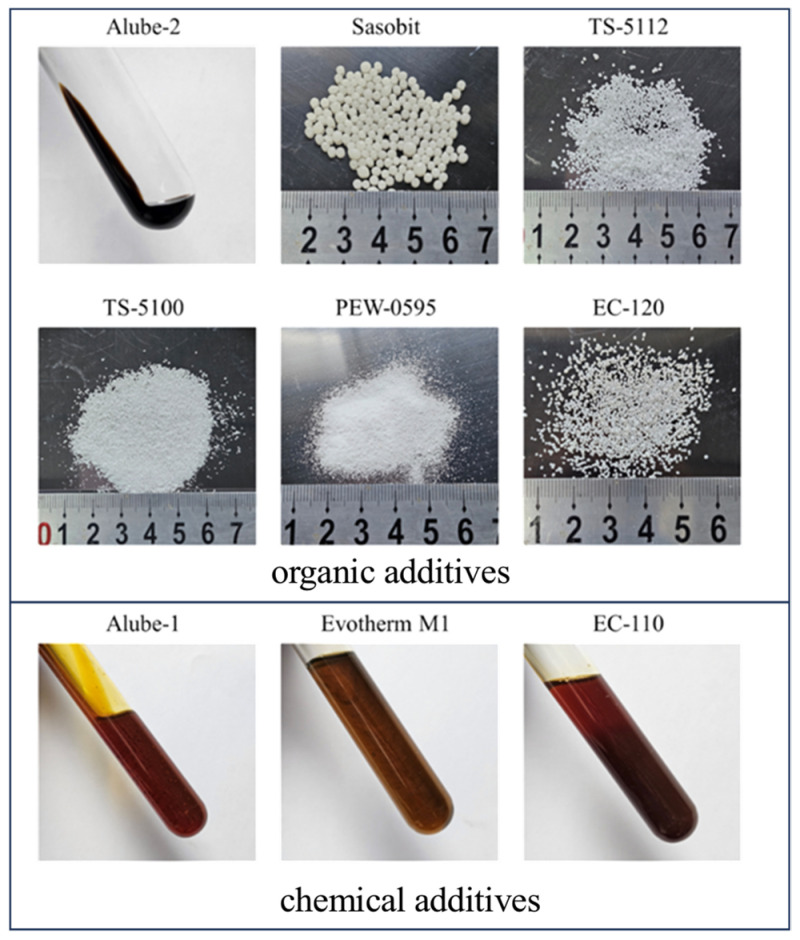
Morphology of WMA additives.

The basic technical properties of locally available base asphalt binder (grade 70#, hereinafter referred to 70#JZ) used in this study are presented in Table 1. Nine typical WMA additives currently on the market, comprising six organic additives (Sasobit, TS-5100, TS-5112, EC-120, PEW-0595 and Alube-2) and three chemical additives (Evotherm M1, EC-110, and Alube-1), were selected for this study, as displayed in Figure 1. Their basic properties and dosages, according to the manufacturers’ recommendations, are presented in Table 2. Basic properties, like penetration value, softening point and ductility, for WMA additive-modified binders (asphalt sample name abbreviated as additive name) with recommended median dosage are also presented in Table 1.

### 2.2. Preparation of WMA Additive-Modified Asphalt Binders

**Figure 2:**
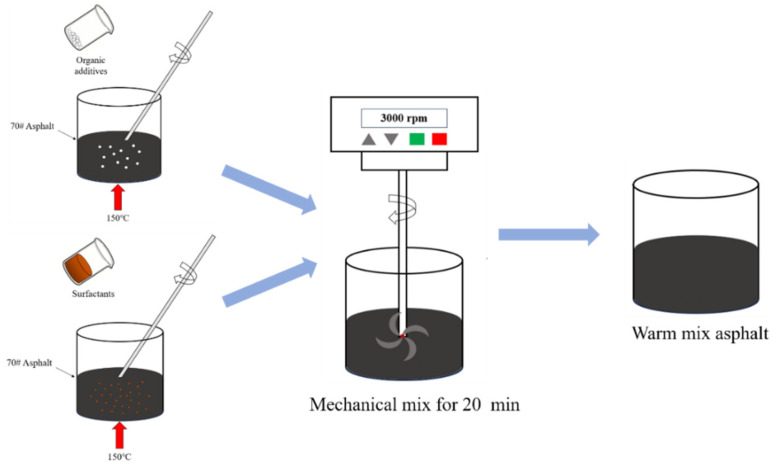
Preparation process of WMA additive-modified asphalt binders.

70#JZ asphalt was first heated to approximately 150 °C to ensure sufficient flowability for mixing with WMA additives. Then, each of the nine WMA additives was mixed with 70#JZ asphalt at the dosage of the manufacturers’ recommendation, as presented in Table 2. To ensure a uniform blending, each WMA additive was mixed with 70#JZ asphalt binder by a high-speed shear mixer at 3000 rpm for 20 min. The preparation process for WMA additive-modified asphalt binder is presented in Figure 2. They were subjected to short-term RTFOT and long-term PAV aging for later testing, including DSR and BFE tests.

### 2.3. The Test Method

#### 2.3.1. The Rotational Viscosity (RV) Test

Viscosity is a physical quantity that describes a fluid’s resistance to flow. Viscosity is an important mechanical index of asphalt binder, measuring its resistance to permanent deformation [20]. Meanwhile, it is also a good parameter for quantifying the viscosity-reduction effect of WMA additives on asphalt binders.

In this study, NDJ-1D Brookfield viscometer manufactured by Shanghai Fangrui Company was employed to quantify the torque required to maintain a constant rotational speed of the cylindrical spindle at temperatures between 115 °C to 155 °C with 10 °C intervals. This torque in turn can be used to determine the viscosity of warm-mix asphalt binder. The detailed testing procedures can be referred to AASHTO T 316 [21] and three samples for each test were evaluated.

#### 2.3.2. The Temperature Sweep Test

According to AASHTO T 315 [22], a temperature sweep test was conducted on previously prepared warm-mix asphalt binders at temperatures ranging from 46 °C to 82 °C with 6 °C intervals. The test was carried out under the conditions of 1.59 Hz and 12% strain. In this study, rheological properties, including phase angle (δ), complex modulus (G*), and rutting factor (G*/sinδ), were evaluated. It should be noted that three samples for each test were examined.

#### 2.3.3. The Multi-Stress Creep Recovery (MSCR) Test

The MSCR test was developed to simulate the nonlinear rheological behavior of asphalt binders. The test was conducted using a Dynamic Shear Rheometer (DSR) in creep mode at a specified temperature, i.e., 64 °C in this study. The test was performed under a constant stress creep for a 1.0 s duration and a 9.0 s rest period for two loading levels (0.1 kPa and 3.2 kPa). The detailed testing procedures and data process method can be referred to as AASHTO T 350 [23], and three samples for each testing condition were evaluated. The commonly obtained three parameters, average percent recovery (R), non-recoverable creep compliance (Jnr)and percent difference in non-recoverable creep compliance (Jnr-diff), can be determined using Equations (1), (2) and (3), respectively. It should be noted that the temperature sweep and the MSCR test were conducted on DSR. This was a Discovery HR-10 from TA, and both tests were performed using a 25 mm plate loading mode with a 1 mm gap.

(1) $$R=\frac{\sum_{i=1}^{10}\left(\frac{\varepsilon_{1}-\varepsilon_{10}}{\varepsilon_{1}}\right)}{10}\times 100$$

(2) $$J_{nr}=\frac{\sum_{i=1}^{10}(\frac{\varepsilon_{10}}{\tau }{)}_{i}}{10}$$

(3) $$J_{nr-diff}=\frac{\left[J_{nr3.2}-J_{nr0.1}\right]\times 100}{J_{nr0.1}}$$

where *R* = average percent recovery for 10 loading and recovery cycles at the stress level of τ; *ε*_1_ = induced stain at the end of each loading cycle; *ε*_10_ = residual strain at the end of the corresponding recovery period; *J_nr_* = average non-recoverable creep compliance at the stress level of τ; τ = the stress level, set to 0.1 kPa and 3.2 kPa; *J_nr-diff_* = the percent difference in non-recoverable creep compliance between 0.1 kPa and 3.2 kPa.

#### 2.3.4. The Binder Fracture Energy (BFE) Test

**Figure 3:**
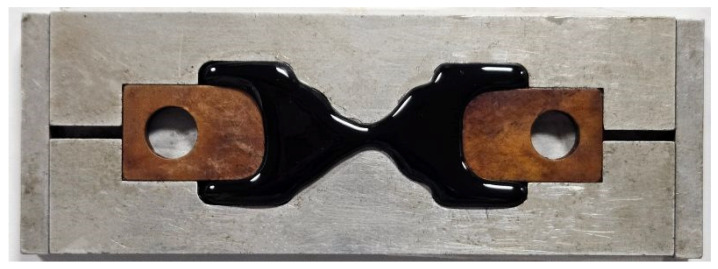
Typical BFE test sample.

**Figure 4:**
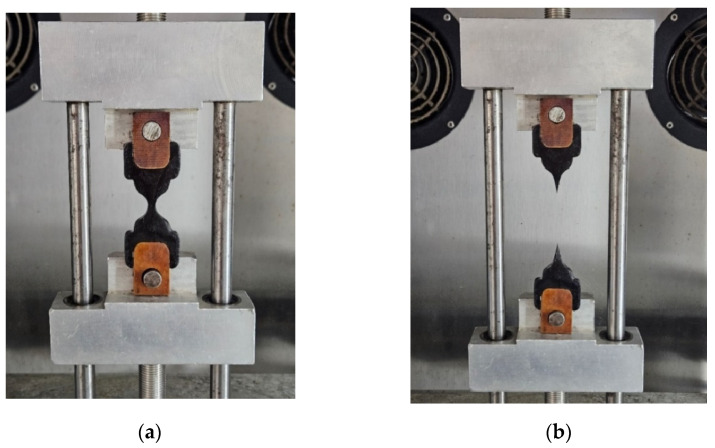
BFE test setup before testing (**a**) and after testing (**b**).

Fracture energy density (FED), defined as the maximum energy that can be stored in a unit volume of material before fracture, can be determined from the binder fracture energy (BFE) test according to AASHTO TP 127 [24]. FED is a reliable parameter for evaluating binder cracking resistance at intermediate temperatures [25]. This test was conducted at 15 °C, and the monotonic loading rate was 500 mm/min. The typical BFE sample can be seen in Figure 3. The BFE test setup is presented in Figure 4, (a) before testing and (b) after failure. The true stress and true strain before the first peak, geometry at the first stress peak, and the true stress and true strain after the first stress peak can be calculated using Equations (4), (5) and (6), respectively, which was used to determine FED for warm-mix asphalt binders. The detailed testing procedures and data process methods can be AASHTO TP 127 [24], and Chen et al. [25], and three samples for each test were examined.

(4) $$\sigma =\frac{F}{A}=\frac{F}{0.006x^{2}-0.925x+24};\varepsilon =0.0006x^{2}+0.0537x$$

(5) $$A_{peak}=0.006x_{peak}^{2}-0.925x+24;L_{peak}=0.0086x_{peak}^{2}+0.132x+3$$

(6) $$\sigma =\frac{F}{A_{1}}=\frac{F}{A_{peak}\times \frac{L_{peak}}{L_{1}}};\varepsilon =\ln\left(\frac{L_{1}}{L_{peak}}\right)+\varepsilon_{Before}$$

where

A_1_ = center cross-sectional area after the first stress peak (mm^2^),

L_1_ = length of the initial 3 mm middle section after the first stress peak (mm),

$\varepsilon_{Before}$ = true strain before the first stress peak, and

x = specimen extension in the length direction.

**Figure 5:**
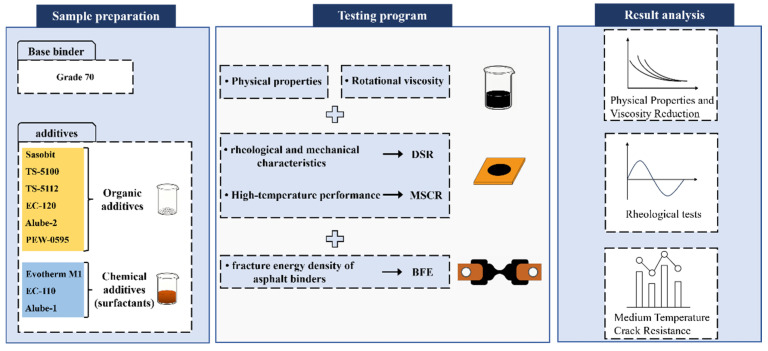
Flow chart of experimental work.

The research plan of this study is presented in Figure 5.

## 3. Results

### 3.1. Viscosity-Reducing Effect

**Figure 6:**
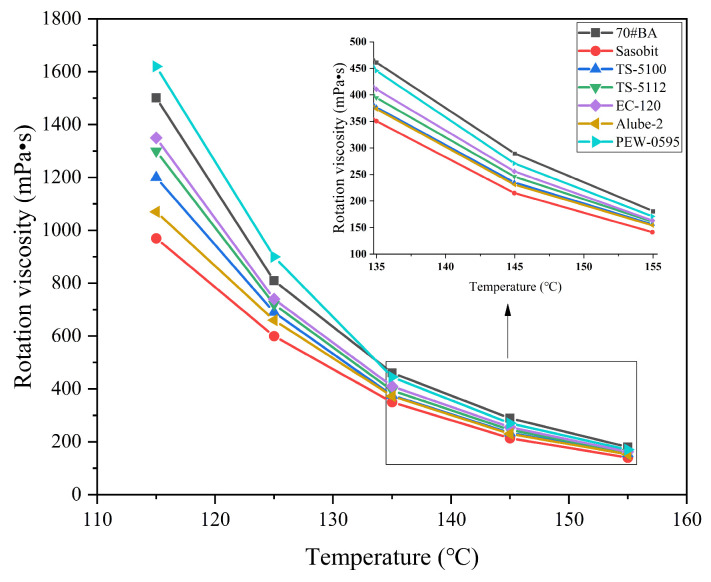
Rotational viscosity for organic additive-modified asphalt binders.

As stated earlier, asphalt binder viscosity has been used to determine the mixing and compaction temperatures during asphalt pavement construction process. Thus, the viscosity test results for all nine warm-mix asphalt binders were chosen to evaluate the reduction effects of WMA additives on binder viscosity. The rotational viscosity results for organic additive-modified binders are presented in Figure 6.

As expected, the viscosity of each asphalt binder was certainly reduced with the temperature increase, as illustrated in Figure 6. It should be noted that the viscosity of all warm-mix asphalt binders was lower than 70#JZ asphalt binder in the temperature of 115 °C to 155 °C, except for PEW-0595. For PEW-0595, its viscosity was lower than that of 70#JZ above 135 °C. However, its viscosity was higher than 70#JZ asphalt below 135 °C. The melting point of Fischer–Tropsch wax is 110 °C to 120 °C, which was the primary component of WMA additives like Sasobit, TS-5100, and TS-5112. The melting of Fischer–Tropsch wax was responsible for the viscosity reduction in asphalt binders modified with organic WMA additives. In contrast, the melting point of the oxidized polyethylene wax is approximately 130 °C, which was the main ingredient for PEW-0595. Approximately 110 °C to 135 °C, the viscosity of PEW-0595 was increased as long as the oxidized polyethylene wax remained in the form of semi-solid or solid materials. Once the temperature was raised beyond 135 °C, melting the oxidized polyethylene wax reduced the asphalt binder’s viscosity, as shown in Figure 6. It should also be noted that the Sasobit additive has the best viscosity-reduction effect compared to other organic WMA additives. After completely melting WMA additives at high temperatures beyond 135 °C, the differences in viscosity reduction were significantly reduced.

**Figure 7:**
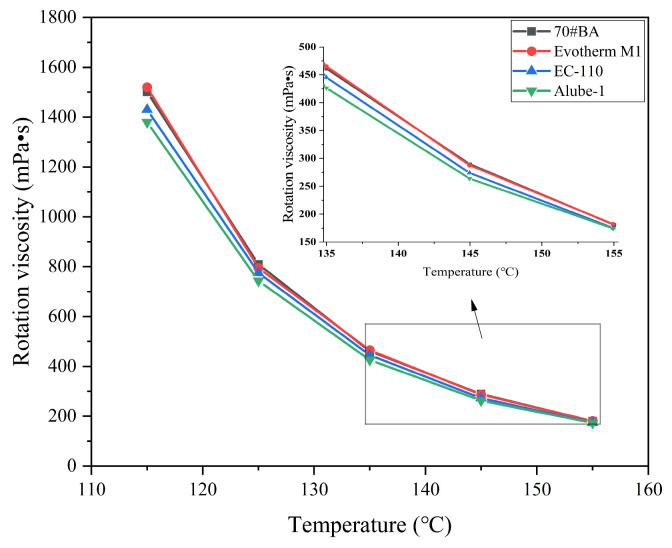
Rotational viscosity for chemical additive-modified asphalt binders.

The viscosity of asphalt binders prepared with chemical additives is presented in Figure 7. The viscosity of asphalt binders modified with chemical additives was slightly lower than that of 70#JZ asphalt binder at 115 °C to 155 °C. Overall, it can be concluded that chemical WMA additives have a negligible effect on the viscosity of asphalt binder. Since surfactant-type WMA additives can enhance the interface interaction between aggregate and asphalt binders [26], it was usually added less than 1% by the weight of asphalt binders.

It can be concluded that organic additives generally provide a viscosity-reduction effect due to their wax components melting at high temperatures. On the other hand, the chemical additives, mainly surfactants, improve lubrication between the asphalt binder and aggregate without affecting binder viscosity [27], thereby reducing the mixing and compaction temperatures of the asphalt pavements.

### 3.2. High-Temperature Rutting Resistance

#### 3.2.1. Temperature Sweep Tests

**Figure 8:**
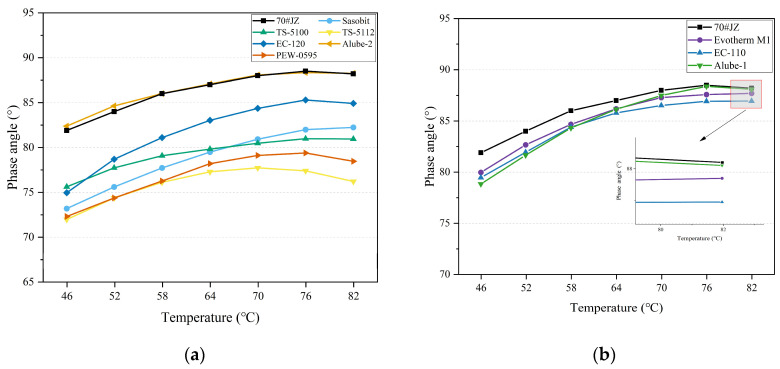
Phase angle for warm-mix asphalt binders with WMA additives: (**a**) organic additives and (**b**) chemical additives.

The phase angles (δ) of warm-mix asphalt binders for organic and chemical additives were presented in Figure 8a and Figure 8b, respectively. As expected, δ values increased with temperature increase for organic and chemical warm-mix asphalt binders. It should be pointed out that a peak value of δ was also observed at approximately 76 °C. Before this peak value of δ, shear thinning behavior can be observed with the temperature increase. On the other hand, asphalt binders exhibited shear thickening behavior after the peak, probably due to the rearrangement of internal structures.

Further, except Alube-2, warm-mix asphalt binders with organic additives exhibited lower δ than70#JZ asphalt, as seen in Figure 8a. It should be remembered that organic additives are either solid or semi-solid in the temperature range of 46–82 °C, which is well below their melting points. The primary function of organic additives is to reinforce the asphalt binders in this temperature range. For warm-mix asphalt binders with chemical additives, a slight reduction in δ can be observed throughout the testing temperature range, as seen in Figure 8b. This indicated that surfactant-type WMA additives have negligible effects on δ of asphalt binder, consistent with rotational viscosity results as presented in Figure 7.

**Figure 9:**
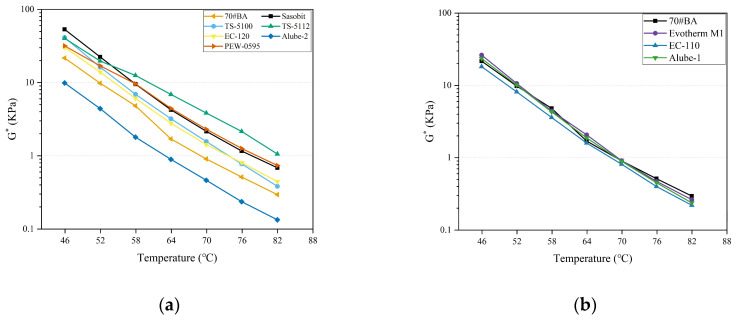
Complex modulus for warm-mix asphalt binders with WMA additives: (**a**) organic additives and (**b**) chemical additives.

Figure 9a and Figure 9b present the complex modulus (G*) of warm-mix asphalt binders with organic and chemical additives, respectively. It can be found that G* was decreased with the increase in temperatures for both warm-mix asphalt binders with organic and chemical additives. For all warm-mix asphalt binders with organic additives, except Alube-2, the strengthening effect was again exhibited in higher G* than 70#JZ asphalt. This was similar to what was reported in the δ results in Figure 8. On the other hand, the strengthening effect was trivial for warm-mix asphalt binders with chemical additives compared to 70#JZ asphalt. Again, it confirmed that the mechanism of chemical additives in reducing the mixing and compaction temperatures was to act at the microscopic interface, decreasing the frictional forces between aggregates and asphalt binders [14].

**Figure 10:**
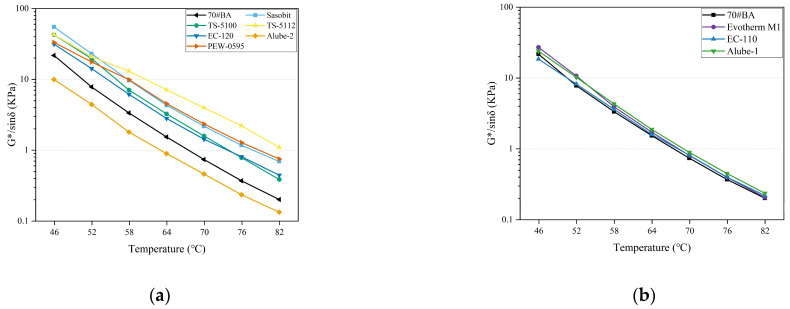
Rutting factor for warm-mix asphalt binders with WMA additives: (**a**) organic additives and (**b**) chemical additives.

Rutting factor (G*/sinδ) has been widely used to evaluate the rutting resistance of asphalt binders [17]. Figure 10a and Figure 10b present the G*/sinδ of warm-mix asphalt binders with organic and chemical additives, respectively. With the increased temperature, G*/sinδ for warm-mix asphalt binders with organic and chemical additives decreased, indicating lower rutting resistance with elevated temperatures. In particular, all organic additives except Alube-2 displayed beneficial effects on the rutting resistance of asphalt binders. This was probably due to the interaction between waxes and asphalt binder, which created a rigid molecular network within the binder. Long-chain aliphatic hydrocarbons assisted in the rigid molecular chains interconnection. Once again, the effect of chemical additives on the rutting resistance of asphalt binders can be neglected since it acts as a lubricant in the asphalt–aggregate contact area and enhances the performance of asphalt-coating adhesion on aggregate surfaces [28].

#### 3.2.2. MSCR Tests

**Figure 11:**
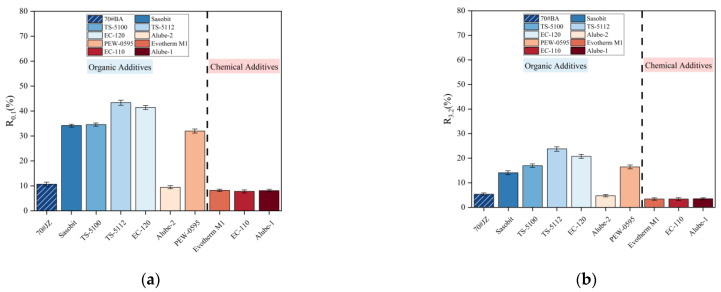
Percent recovery for warm-mix asphalt binders with WMA additives under different loading levels at 64 °C: (**a**) 0.1 kPa; (**b**) 3.2 kPa.

As stated earlier, the MSCR test was developed to replace the rutting factor (G*/sinδ) since it was considerably more discriminating in identifying the rutting potential of modified binders. Figure 11a,b present R0.1 and R3.2, percent recovery (R) results of MSCR for warm-mix asphalt binders under 64 °C, were presented in Figure 11a and Figure 11b, respectively. It can be seen from Figure 11 that when the stress level was increased from 0.1 kPa to 3.2 kPa, R was considerably reduced, which indicated a stronger elastic response under a lower loading level. Figure 11 also shows that heavy loading makes asphalt binders more susceptible to rutting damages.

Further, all warm-mix asphalt binders with organic additives, except Alube-2, showed much higher R than 70#JZ asphalt under different loading levels. It clearly indicates rutting resistance enhancement for organic additives except Alube-2 in this study. For chemical additives, a slight reduction of percent recovery as compared with 70#JZ asphalt can be observed. Warm-mix asphalt binders with chemical additives showed relatively lower R than warm-mix asphalt binders with organic additives, which indicated its lower rutting resistance for surfactant-type additives. R is a direct demonstration of the stiffness of asphalt binders. The findings from the results of R are consistent to that of G* as shown in Figure 9.

**Figure 12:**
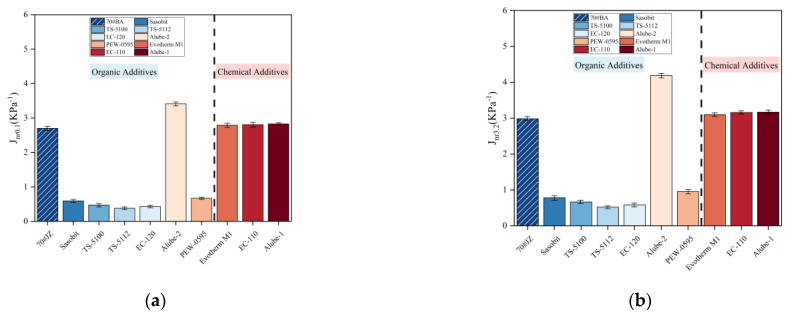
Non-recoverable creep compliance for warm-mix asphalt binders with WMA additives under different loading levels at 64 °C: (**a**) 0.1 kPa; (**b**) 3.2 kPa.

Figure 12a and Figure 12b present non-recoverable creep compliance (Jnr) results of MSCR, including the Jnr0.1 and Jnr3.2 for warm-mix asphalt binders under 64 °C, respectively. Interestingly, Jnr was only slightly increased when the loading level was increased from 0.1 kPa to 3.2 kPa as shown in Figure 12a,b. Further, the lower Jnr value for warm-mix asphalt binders with organic additives than that of 70#JZ asphalt indicated lower permanent deformation and, thus, better rutting resistance. Slightly higher but negligible Jnr for warm-mix asphalt binders with chemical additives than 70#JZ asphalt again demonstrated that it has very trivial effects on binder properties. Significant enhancement of rutting resistance for organic additives but negligible effect on rutting resistance for chemical additives based on MSCR tests have also been reported by other researchers [29,30].

**Figure 13:**
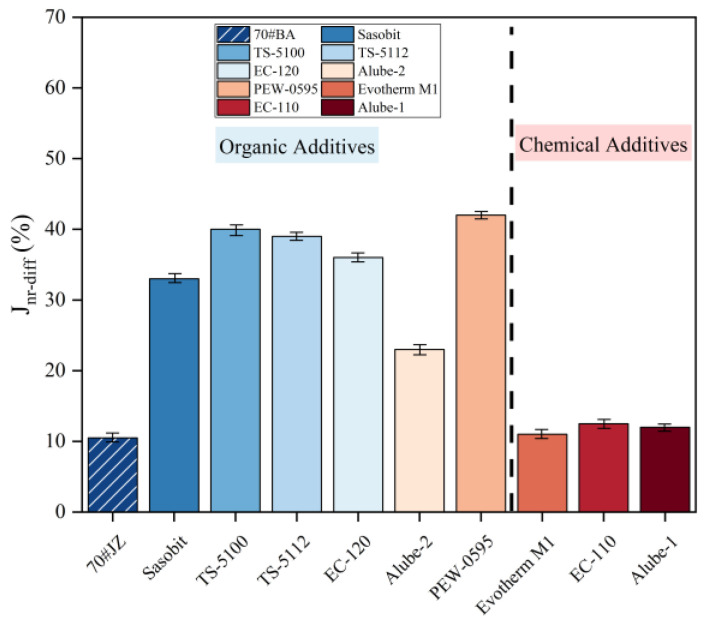
Percent difference in non-recoverable creep for warm-mix asphalt binders.

As a measurement of stress sensitivity, the percent difference in non-recoverable creep compliance (Jnr-diff) values for warm-mix asphalt binders was presented in Figure 13.

It can be seen that Jnr-diff values were all well below the proposed limit of 75%, as stated in AASHTO M332 [31]. However, adding WMA additives increased the stress sensitivity level compared to 70#JZ asphalt. Meanwhile, Fischer–Tropsch wax and polyethylene wax from organic additives increased the stress sensitivity level of asphalt binders. In contrast, chemical additives exert a minimal influence on the stress sensitivity of the binder, which may be attributed to their little-to-nothing effect on asphalt binders.

All in all, it can be found from the temperature sweep test and MSCR test results that the organic additives have a more positive effect on the rutting resistance of asphalt binder than that of chemical additives.

### 3.3. BFE Tests

**Figure 14:**
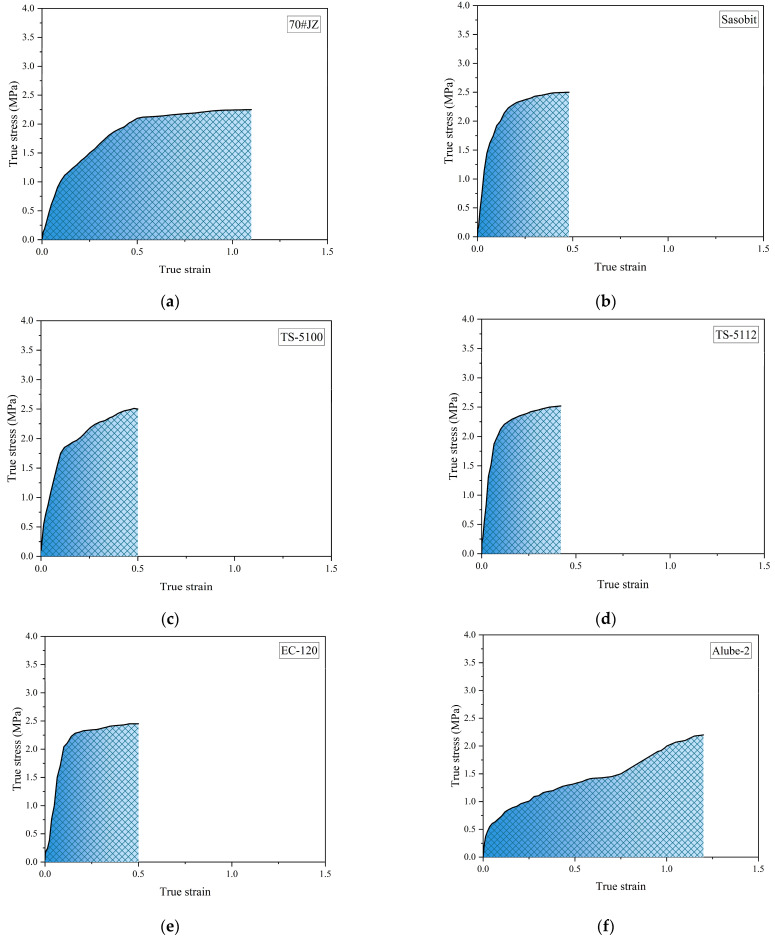

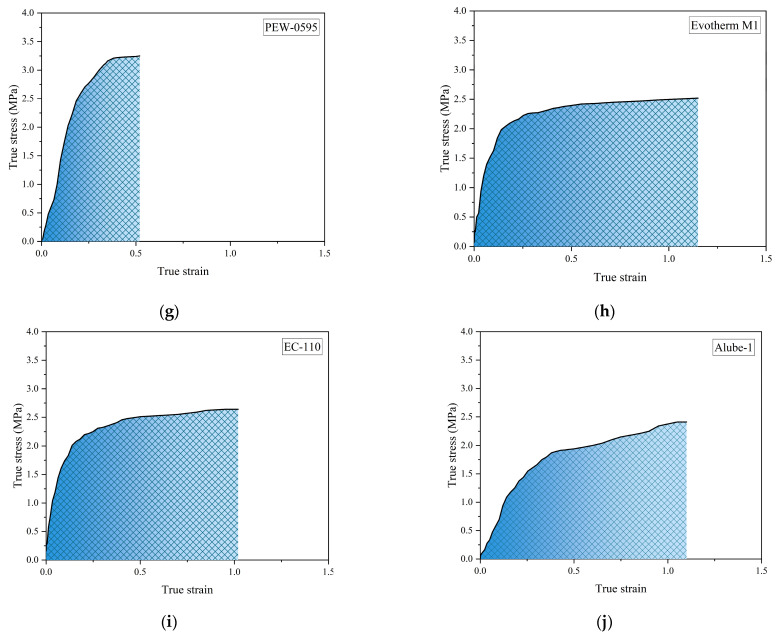
True stress–true strain curves for warm-mix asphalt binders: 70#JZ (**a**); Sasobit (**b**); TS-5100 (**c**); TS-5112 (**d**); EC-120 (**e**); Alube-2 (**f**); PEW-0595 (**g**); Evotherm M1 (**h**); Ec-110 (**i**); Alube-2 (**j**).

In addition rutting resistance, intermediate cracking resistance is also very critical for the performance of asphalt binders. The true stress and true strain for asphalt binders are presented in Figure 14.

It can be seen that warm-mix asphalt binders with organic additives, except for Alube-2, all have a failure strain of approximately 0.5, as shown in Figure 14b–e, which is significantly lower than that of 70#JZ asphalt (Figure 14a). This is consistent with the increased G*, which strongly indicates increased brittleness. Accordingly, the higher brittleness of warm-mix asphalt binders with organic additives than 70#JZ asphalt will eventually lead to lower cracking resistance. By contrast, warm-mix asphalt binders with chemical additives showed similar failure strain values to 70#JZ asphalt, as displayed in Figure 14h–j. This demonstrates that chemical additives have minimal effects on the corresponding binder properties. Despite the low rutting resistance for Alube-2, it has exhibited exceedingly higher failure strain, as displayed in Figure 14f, which can be characterized as ductile failure.

**Figure 15:**
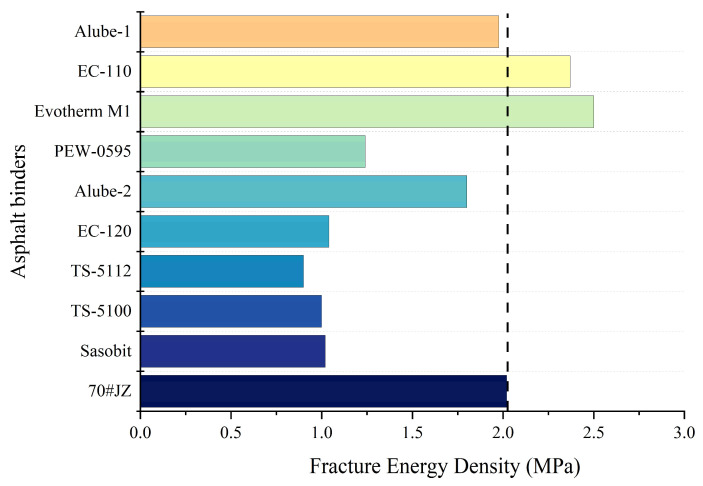
Fracture energy density for warm-mix asphalt binders.

FED, determined as the area under the true stress–true strain curve, was calculated and presented in Figure 15. The exceeding low failure strain of TS-5112, shown in Figure 14d, leads to the lowest FED as compared with 70#JZ asphalt and other warm-mix asphalt binders. As stated earlier, all warm-mix asphalt binders with organic additives except Alube-2 have higher G* than 70#JZ asphalt. The combination of higher stiffness, i.e., significantly lower failure strain and comparable strength with 70#JZ asphalt, leads to their much lower FED than 70#JZ asphalt. For warm-mix asphalt with chemical additives, comparable (Alube-1) or slightly better cracking resistance than 70#JZ asphalt can also be observed. This again confirmed that chemical additives have minimal effects on the performance of asphalt binders.

It should be noted that the improved rutting resistance of warm-mix asphalt binders with organic additives was due to the reinforcing effects of wax in the testing temperature range. However, its cracking resistance at intermediate temperatures was compromised due to the increased stiffness. This contradiction between rutting resistance at high temperatures and cracking resistance at intermediate temperatures is worth noting in the future when selecting warm-mix additives, especially in areas with concerns about cracking resistance.

## 4. Conclusions

This study evaluated two categories of warm-mix additives, including six organic and three chemical additives, for their effects on asphalt binder properties, emphasizing rutting and cracking resistance. The following conclusions can be made:

(1) Wax component type and the corresponding melting point of organic additives were critical for the viscosity-reduction effect, rutting resistance, and cracking resistance on asphalt binders.

(2) The viscosity-reduction effect of warm-mix asphalt binders with organic additives was significantly observed. However, chemical additives were observed to have a viscosity-reducing minimal impact on the viscosity of asphalt binders.

(3) Since the wax component of organic additives remained semi-solid or solid, it mainly worked as a reinforcing agent at high pavement service temperatures. A contradiction existed between increased rutting resistance and decreased cracking resistance of warm-mix asphalt binders with organic additives.

(4) Chemical additives have a minimal effect on the asphalt binder properties at both high and intermediate service temperatures, given that asphalt mixture workability and compactibility were increased by enhancing the microscopic interface interaction between aggregates and asphalt binders.

In the future, the low-temperature cracking resistance of warm-mix asphalt binders should be well investigated, which can help build a more comprehensive overview of the effects of warm-mix additives on the corresponding binder properties. Meanwhile, the impact of molecular weight and structure on organic additives should be further studied. This can help with the application and development of organic additives. In addition, efforts should be made to determine the application conditions for specific additives under different climatic conditions, which can help to realize the full potential of warm-mix additives.

## Figures and Tables

**Table 1:** Properties of different WMA with the recommended median dosage.

Type	Penetration Value/0.1 mm	Softening Point/°C	Ductility at 10 °C/cm
70#JZ	67	47.5	31.5
Alube-2	71	45	32.7
Sasobit	49	70.2	7.8
TS-5112	43	71.5	6.3
TS-5100	46	63.7	6.5
PEW-0595	47	68.4	6.9
EC-120	45	67.5	7.4
Alube-1	64	47.1	24.9
Evotherm-M1	64	47.6	25.8
EC-110	65	48.2	24.5

**Table 2:** Basic properties of WMA additives and the corresponding dosage by weight of asphalt binders.

WMA Additive Type	Physical State	Color	Molecular Weight (g/mol)	Melting Point (°C)	Dosage (%)	Manufacturer
Alube-2	Liquid	Black	-	-	5	Xinli Highway Material Co., (Chengdu, China)
Sasobit	Solid particle	Off-white to pale brown	Approx. 1000	100–110	3	Sasol-Wax Co., (Hamburg, Germany)
TS-5112	Solid powder	White	1300–1500	109–115	3	Tianshi Material Technology Co., (Nanjing, China)
TS-5100	Solid powder	White	700–1100	102–107	3	Tianshi Material Technology Co., (Nanjing, China)
PEW-0595	Solid powder	White	1500–5000	137	3	Tianshi Material Technology Co., (Nanjing, China)
EC-120	Tiny tablet and granular solid	White	Approx. 1000	100–110	3	Haichuan Material Technology Co., (Shenzhen, China)
Alube-1	Liquid	Brown	-	-	0.5	Xinli Highway Material Co., (Chengdu, China)
Evotherm M1	Liquid	Dark amber liquid	-	-	0.8	Ingevity Co., (Zhuhai, China)
EC-110	Liquid	Brown	-	-	0.3	Haichuan Material Technology Co., (Shenzhen, China)

## Data Availability

The original contributions presented in this study are included in the article. Further inquiries can be directed to the corresponding author.

## References

[1] Sukhija M., Saboo N. (2020). A comprehensive review of warm mix asphalt mixtures-laboratory to field. Constr. Build. Mater..

[2] Cheraghian G., Cannone Falchetto A., You Z., Chen S., Kim Y.S., Westerhoff J., Moon K.H., Wistuba M.P. (2020). Warm mix asphalt technology: An up to date review. J. Clean. Prod..

[3] Rubio M.C., Martínez G., Baena L., Moreno F. (2012). Warm mix asphalt: An overview. J. Clean. Prod..

[4] Ghabchi R., Rani S., Zaman M., Ali S.A. (2021). Effect of WMA additive on properties of PPA-modified asphalt binders containing anti-stripping agent. Int. J. Pavement Eng..

[5] Şahan N., Kumandaş A., Kabadayı E., Çavdar E., Oruç Ş. (2023). The use of wax-based additives in bitumen modification: A systematic quantitative literature review. Constr. Build. Mater..

[6] Canestrari F., Graziani A., Valter P., Bahia H.U. (2013). Rheological properties of bituminous binders with synthetic wax. Int. J. Pavement Res. Technol..

[7] Jamaloei M.H., Esfahani M.A., Torkaman M.F. (2019). Rheological and mechanical properties of bitumen modified with sasobit, polyethylene, paraffin, and their mixture. J. Mater. Civ. Eng..

[8] Zhang K., Luo Y., Chen F., Han F. (2020). Performance evaluation of new warm mix asphalt and water stability of its mixture based on laboratory tests. Constr. Build. Mater..

[9] Yang S., Yan K., He W., Wang Z. (2019). Effects of Sasobit and Deurex additives on asphalt binders at midrange and high temperatures. Int. J. Pavement Eng..

[10] Yuan H., Liu J., Ding H., Xie Q., Qiu Y. (2024). Evaluation of physical hardening of wax-based warm mix asphalt binders from low-temperature rheological properties. Constr. Build. Mater..

[11] Yu H., Chen Q., Lin Y., Dong N. (2023). Effect of wax additives on asphalt rheological behavior as road paving material. Mater. Today Commun..

[12] Zelelew H., Paugh C., Corrigan M., Belagutti S., Ramakrishnareddy J. (2013). Laboratory evaluation of the mechanical properties of plant-produced warm-mix asphalt mixtures. Road Mater. Pavement Des..

[13] Tasdemir Y. (2009). High temperature properties of wax modified binders and asphalt mixtures. Constr. Build. Mater..

[14] Pereira R., Almeida-Costa A., Duarte C., Benta A. (2018). Warm mix asphalt: Chemical additives’ effects on bitumen properties and limestone aggregates mixture compactibility. Int. J. Pavement Res. Technol..

[15] Bazzaz M., Darabi M.K., Little D.N., Garg N. (2019). Effect of Evotherm-M1 on properties of asphaltic materials used at NAPMRC testing facility. J. Test. Eval..

[16] Luo J., Yang Y., Huang W., Xie C., Chen J., Liu H., Ren T., Huang X. (2022). Physical, rheological, and microsurface characteristics of high-viscosity binder modified with WMA agents. Adv. Mater. Sci. Eng..

[17] Jamshidi A., Golchin B., Hamzah M.O., Turner P. (2015). Selection of type of warm mix asphalt additive based on the rheological properties of asphalt binders. J. Clean. Prod..

[18] Wagh V.P., Sukhija M., Gupta A. (2023). Exploring the consequences of reduced aging on the performance of warm mix asphalt binders. Int. J. Pavement Eng..

[19] Wu S., Zhang W., Shen S., Li X., Muhunthan B., Mohammad L.N. (2017). Field-aged asphalt binder performance evaluation for Evotherm warm mix asphalt: Comparisons with hot mix asphalt. Constr. Build. Mater..

[20] Pandey A., Singh S.K., Islam S.S., Ransingchung R. N. G.D., Raju S., Ravindranath S.S. (2023). Rheological analysis of performance grade rutting and fatigue cracking criteria in asphalt binders. Int. J. Pavement Res. Technol..

[21] (2019). Standard Method of Test for Viscosity Determination of Asphalt Binder Using Rotational Viscometer.

[22] (2020). Standard Method of Test for Determining the Rheological Properties of Asphalt Binder Using a Dynamic Shear Rheometer (DSR).

[23] (2019). Standard Method of Test for Multiple Stress Creep Recovery (MSCR) Test of Asphalt Binder Using a Dynamic Shear Rheometer (DSR).

[24] (2019). Determining the Fracture Energy Density of Asphalt Binder Using the Binder Fracture Energy (BFE) Test.

[25] Chen Y., Dong S., Wang H., Gao R., You Z. (2020). Using surface free energy to evaluate the fracture performance of asphalt binders. Constr. Build. Mater..

[26] Yang X., You Z., Hasan M.R.M., Diab A., Shao H., Chen S., Ge D. (2017). Environmental and mechanical performance of crumb rubber modified warm mix asphalt using Evotherm. J. Clean. Prod..

[27] Sol-Sánchez M., Moreno-Navarro F., Rubio-Gámez M.C. (2017). Study of surfactant additives for the manufacture of warm mix asphalt: From laboratory design to asphalt plant manufacture. Appl. Sci..

[28] Shi J., Fan W., Wang T., Zhao P., Che F. (2020). Evaluation of the physical performance and working mechanism of asphalt containing a surfactant warm mix additive. Adv. Mater. Sci. Eng..

[29] Kumar R., Saboo N., Kumar P., Chandra S. (2017). Effect of warm mix additives on creep and recovery response of conventional and polymer modified asphalt binders. Constr. Build. Mater..

[30] Bennert T., Reinke G., Mogawer W., Mooney K. (2010). Assessment of Workability and Compactability of Warm-Mix Asphalt. Transp. Res. Rec..

[31] (2023). Standard Specification for Performance-Graded Asphalt Binder Using Multiple Stress Creep Recovery (MSCR) Test.

